# The Royal British Nurses' Association Club

**Published:** 1921-05-28

**Authors:** 


### The Royal British Nurses' Association Club.

### OPENING BY H.R.H. PRINCESS CHRISTIAN.

The Headquarters and Club of the Royal British Nurses' Association at 194 Queen's Gate, S.W., were opened on Wednesday, May 18. The premises are those occupied by Queen Mary's Hostel for Nurses during the war, and through the generosity of Sir Harold Boulton and the Committee of Queen Mary's Hostel the whole of the furniture and equipment have been handed over to the Association as a free gift. The house is a handsome one of six floors, with large and beautifully proportioned public rooms, and sleeping accommodation for some thirty-six members. Already ten are in residence, although the house was only taken over by the Royal British Nurses' Association on April 15 last.

The house is comfortably furnished and equipped. The ceilings and mantelpieces in the public rooms are after Adam, the floors are paraquet, with many Persian rugs, and there is a Waygood passenger lift from the basement. There is a continuous supply of hot water for baths (for which no charge is made), and hot and cold water and a lavatory on every floor. The long corridors provide space and air, and the ample cupboard accommodation will be a joy to all. The kitchens are in the basement, and their black-and-white-tiled floors and white-tiled walls throughout ensure the maximum of cleanliness at the minimum of labour and cost.

The public rooms on May 18 were beautifully decorated with a gay profusion of flowers given by the Registered Nurses' Parliamentary Council, the Registered Nurses' Society, and individual members and friends. H.R.H. Princess Christian, who has been President of the Association since its incorporation, was accompanied by H.H. Princess Marie Louise. She was received by the Executive and honorary officers, and the opening ceremony took place in the Secretary's office. Here, after prayers by the Sub-Dean of the Chapels Royal, Canon Edgar Sheppard, and speeches by H.R.H. and Miss Heather-Bigg, Vice-Chairman, the Princess fixed the brass commemorative tablet recording the taking over of the house by the Association and its opening by herself, which pairs with a similar tablet recording the gift of £2,000 from the Australian Red Cross Society towards the equipment of Queen Mary's Hostel. Princess Christian recalled the peculiar pleasure she feels in carrying the scheme for founding a Club to a successful conclusion, due largely to the co-operation and loyalty of the members of the Association. She thanked the members for their part in establishing the Club, and Sir Harold Boulton and his Committee for their generous gift, and expressed her pleasure at the intention of the nurses to raise an Endowment Fund for the principal room in the Club and to connect their President's name with it. She thanked Miss Macdonald, the valued Secretary of the Association, for her devoted untiring services. H.R.H. has had ample opportunity to appraise these services, extending over nearly twelve years, for, as Miss Heather-Bigg later said, the Princess has been no mere figure-head in the Association, but has always taken a keen interest in it.

### Terms for Membership.

Membership of the Club is not confined to members of the Royal British Nurses' Association, but it is confined for the present to members of the nursing profession. Members of the R.B.N.A. pay an entrance fee of £1 1s., and an annual subscription of £1 1s. for town and 10s. 6d. for country. Non-members pay an entrance fee of £1 10s., and an annual subscription of £2 2s. These are preferential terms, and apply only to those nurses who join before July 16 next. The charges for residence are £2 7s. 6d. weekly for bed, breakfast, and dinner, with a slight increase on this for a separate bedroom; for part of a week the charge is 8s. a day; while bed and breakfast only will be 5s., and bed and dinner 6s. It will be noticed that lunch or afternoon tea is not included in any of these charges. Meals in the restaurant can be had at reasonable prices.

We congratulate the Royal British Nurses' Association and their members on the acquisition of these fine Club premises, which, under the superintendence of Miss Macdonald, the Secretary, and Miss Beatrice Cutler (late assistant matron, St. Bartholomew's Hospital) as home sister should prove a source of comfort and pleasure to many nurses.

